# Editorial

**Published:** 1868-06

**Authors:** 


### Editorial.

American Medical Association.—In the present number of the Examiner, we devote a large amount of space to the record of proceedings of the recent annual meeting of the Association, in Washington. It is copied from the *National Intelligencer* of that city. The meeting was a very pleasant and profitable one, in all respects. The representation was more general from all parts of the country than for several years past; the Committee of Arrangements had provided excellent accommodations, both for the general meetings and those of the several sections; the reports and volunteer papers were more thoroughly considered in the sections; and the social entertainments were abundant and excellent, without intruding on any of the time required for the transaction of business.

The spirit manifested throughout was earnest, affable, and harmonious; vindicating the truly *national* character of the organization, and affording another demonstration that, in matters of science and humanity, there are no sectional lines. We trust the next meeting, which is to be held in New Orleans, will embrace a still larger delegation, and be actuated by the same spirit.

Annual Meeting of the Illinois State Medical Society. —The annual meeting of this Society was commenced in Quincy, May 19th, 1868, and continued two days. About sixty members were in attendance, and the time was devoted wholly to the transaction of appropriate business. Many of the committees, as usual, failed to report. Yet, there was a goodly number of reports and papers presented; they were more carefully prepared, and received a more full and satisfactory consideration than for several years past. Dr. S. T. Trowbridge, of Decatur, was elected President, and Chicago chosen for the next place of meeting. A full record of the proceedings will be found in the next number of the EXAMINER.

---

SAN FRANCISCO MEDICAL SOCIETY.—The physicians of San Francisco have organized a medical society, and have elected the following named officers for the ensuing year: President, Dr. J. P. Whitney; First Vice-President, Dr. S. R. Harris; Second Vice-President, Dr. J. B. Pigné Dupuytren; Recording Secretary, Dr. Henry Gibbons, Jr.; Corresponding Secretary, Dr. T. M. Logan; Treasurer, Dr. A. G. Soule; Librarian, Dr. Wm. T. Garwood; Committee on Admissions, Drs. J. Haine, H. Gibbons, Senior, Thos. Bennett, W. P. Tilden, G. Hewston. The Committee on Admissions have held a meeting in the Twelfth District Court room, for the purpose of examining the credentials of those who desire to be enrolled as members of the society. Already between sixty and seventy physicians have given in their names, and there is little doubt that many more will come forward when the objects of the society are more fully known.

---

VIENNA, May 6, 1868.

The Medical School of Vienna, although less known in our country than that of Paris or London, undoubtedly ranks first in Europe, as regards its course of instruction and its means of illustration. The term of study is five years, the first two being devoted to the elementary branches of medicine and surgery, embracing, also, botany, geology, and comparative anatomy; the remaining three to the practical branches and clinics.

For the study of morbid anatomy, the pathological museum of Prof. Rokitansky is open. *Post mortem* examinations are also performed in the presence of the class. The department devoted to diseases of the skin, and under the direction of Prof. Hebra, is especially interesting. These wards furnished the originals for his series of lithographed plates, illustrating skin diseases. Arrangements for bathing and steaming are provided, so that, when circumstances demand it, patients can be put through more courses than are required in a Turkish bath.

The hospital is built in the form of a square, enclosing a large court which, being laid out in graveled walks and ornamented with fountains, trees, and shrubbery, forms a beautiful park where patients may enjoy and exercise themselves in the open air. The entire hospital is capable of accommodating 2500 patients without crowding.

Ventilation is most carefully provided for, and, on entering the wards, no odor whatever is perceived. Exit for the light gases is obtained by flues passing out from the ceiling, at the ends of the ward. A similar provision below, carries off the heavy gases. Double windows, situated six feet apart, admit fresh air from both sides, and opening somewhat above the beds, prevent a direct current of air from striking the patient. The beds are placed in single rows on either side, while the centre is free and open. All parts of the building are thoroughly whitewashed several times a year. Erysipelas and gangrene are strangers in this hospital, and the healthy condition of wounds, and ready union after surgical operations, speak plainer than words of the purity and cleanliness of its wards.

Upon the wall, at the head of each patient, is hung a card, giving his name, age, diagnosis, and treatment of his disease, also, the diet he is receiving. The latter is as carefully attended to by the physician as the more direct medication. A complete record of treatment from day to day is thus preserved, for the observation of the student. Few men are emploped in the hospital as nurses, that duty being performed almost entirely by females; they are well trained, and attend to their duties in a manner that cannot be excelled.

Medical clinics are held in the wards, at the bedside, and the students required to diagnose and treat the cases, under the professor. Amputations and all surgical operations, not requiring great skill, are performed by the student, under the direction of the surgeon. This is a practical teaching obtained in but very few schools.

For the study of surgery, diseases of women, laryngoscopy, and dermatology, the student will find Vienna offering very great advantages.

The galvano-caustic is much used. Combining the principles of the ecraseur and actual cautery, it is valuable in operating where hemorrhage is difficult to control.

Besides the General Hospital, Vienna has a large Foundling Hospital, where infants of uncertain parentage are received at any hour, day or night, and, as in the return of stolen property, no questions are asked. The moral influence of such an institution cannot be overestimated. It does not diminish or increase the number of illegitimate children, but it does prevent the destruction of hundreds. I do not think that all the preaching and legislating on the subject of infanticide has yet saved a single young one's life. Here, where provision is made for the reception and care of foundlings, the crime is seldom committed. It is not so much the destruction of the child that the mother desires, as the concealment of her own guilt. When we cannot abolish crime, it is our duty to prevent, as much as possible, its evil results. Experience, in this country, has shown that the foundling hospital is the only successful means of abating infanticide. Yours, truly,

J. S. SHERMAN.

---

THE undersigned beg leave to state that they are no longer connected with the institution called "Hotel for Invalids," on account of its departure from its original plan.

JOS. W. FREER, M.D.
E. C. ROGERS, M.D.
WM. C. LYMAN, M.D.

---

MONEY RECEIPTS TO MAY 27TH.—Drs. Henry Biroth, $3; B. F. Rolfe, 5; F. K. Bailey, 3; R. M. Boyer, 3; D. A. Sheffield, 3; H. C. Hutchinson, 3; Hiram Duncan, 6; Joseph Tefft, 5; Lyman Ware, 3; H. L. Smith, 3; D. S. Jenks, 3; T. Nichols, 3; B. F. Brown, 3; J. A. W. Hostetler, 3; A. Niles, 3; G. R. Bibb, 3; J. T. Frazer, 3; A. M. Maxwell, 3; E. Robbins, 3; R. D. Cogswell, 3.

MORTALITY REPORT FOR THE MONTH OF APRIL:—

### CAUSES OF DEATH.

| | |
|---|---|
| Accident, drowned, | 1 |
| " burns, | 4 |
| " fright, bruises | 2 |
| " fall of floor, | 1 |
| " railroad, | 1 |
| " scalded, | 2 |
| " machinery, | 1 |
| Anæsarca | 1 |
| Angina pectoris, | 1 |
| Aorta, aneurism of | 1 |
| Apoplexy | 4 |
| Atrophy | 1 |
| Birth, premature | 8 |
| " still | 23 |
| " tedious | 1 |
| Bowels, ulceration of, with abdom'l tumor, | 1 |
| Brain, inflammation | 2 |
| " disease of | 1 |
| " congestion of | 2 |
| " œdema of, following pneumo. | 1 |
| " softening of, | 1 |
| Bronchitis | 3 |
| Carries, of spine, | 1 |
| Cancer of breast | 1 |
| " of liver | 1 |
| " of bowels, | 1 |
| " of mouth, | 1 |
| " of uterus, | 2 |
| Cancer, of stomach | 2 |
| Canker sore mouth | 1 |
| Catarrh | 1 |
| Convulsions | 29 |
| Croup | 5 |
| " membranous, | 1 |
| Debility | 5 |
| Diphtheria | 6 |
| Diarrhœa | 1 |
| " chronic, | 2 |
| Dropsy | 1 |
| " chest | 1 |
| " complica'd with cardiac rheu'sm | 1 |
| " of spinal cord, | 2 |
| " from heart dise. | 1 |
| Drowning | 1 |
| Dysentery, | 1 |
| Enteritis | 1 |
| " acute, | 1 |
| Encephalites | 1 |
| Erysipelas, | 2 |
| " neck and face foll. sm.-pox, | 1 |
| Fever, congestive | 1 |
| " puerperal | 5 |
| " remittent | 1 |
| " scarlet | 3 |
| " typhus | 1 |
| " typhoid | 7 |
| Fungus hemadotes | 1 |
| Gastromalacia, | 1 |
| Hemorrhage, secondary | 1 |
| Heart, disease of | 1 |
| " dropsy of, | 1 |
| " " pneumonia | 1 |
| " malformation of | 1 |
| " valvular disease | 3 |
| Hepatitis | 1 |
| Hydrothorax, | 1 |
| Hydrocephalus | 2 |
| " acute, | 2 |
| Inanition | 9 |
| Indigestion | 1 |
| Intemperance | 2 |
| Laryngitis | 3 |
| Liver, tumor of | 1 |
| " induration of | 2 |
| Lungs, congestion of | 3 |
| " emphysema of | 1 |
| " inflammation of | 1 |
| " paralysis of | 1 |
| Manslaughter, | 1 |
| Measles | 7 |
| Metritis, | 1 |
| Meningitis | 5 |
| " cerebro-spinal, | 3 |
| " complic'd with scarlet fever, | 1 |
| " tuberculosis, | 1 |
| Mitral valves, disease, | 1 |
| Nephritis | 1 |
| Nicrosis of inferior mallary bone, | 1 |
| Old Age | 5 |
| " and pneumonia, | 1 |
| " chronic inflammation stomach, | 1 |
| Ovaritis, | 1 |
| Paraphlegia | 1 |
| Paralysis of lungs and heart following bronchitis, | 1 |
| Pericarditis complicated with pneumonia, | 1 |
| " " effusion, | 1 |
| Peritonitis | 1 |
| " metro | 2 |
| Pharyngitis | 1 |
| Phthisis pulmonalisis | 30 |
| Poisoned | 1 |
| Pneumonia | 17 |
| " complicated with measles, | 4 |
| " " teething, | 1 |
| Prostatic calculus and inflam'n of bladder, | 1 |
| Pyæmia | 3 |
| Scurvy and encepaloid cancer of kidneys, | 1 |
| Scrofula | 1 |
| Small-pox | 11 |
| " complicated with pneumonia | 1 |
| Skull, fracture of, | 1 |
| Suicide | 6 |
| Spine, disease of | 1 |
| " fracture of | 1 |
| Syncope from heart disease, | 1 |
| Tabes mesenterica, | 5 |
| Tetanus traumaticus, | 1 |
| " " idiopathic | 1 |
| Tedious labor, | 1 |
| Teething | 6 |
| Tumor, abdominal, | 2 |
| " " and phthisis pulmonalis, | 1 |
| Uræmia of scarlet fever | 1 |
| Varioloid | 1 |
| Whooping-cough | 4 |
| Total | 305 |

Deaths in April, 1868, 305 | Deaths in April, 1867, 278 | Increase, 27

Deaths in March, 1868, 380 | Decrease, 75

### AGES.

| | |
|---|---|
| Under 5 | 144 |
| 5 to 10 | 20 |
| 10 to 20 | 14 |
| 20 to 30 | 30 |
| 30 to 40 | 30 |
| 40 to 50 | 21 |
| 50 to 60 | 18 |
| 60 to 70 | 11 |
| 70 to 80 | 11 |
| 80 to 90 | 3 |
| 90 to 100 | 0 |
| 100 to 110 | 0 |
| Unknown | 3 |
| Total | 305 |

| | | |
|---|---|---|
| Males, 163 | Females, 142 | Total, 305 |
| Single, 204 | Married, 101 | Total, 305 |
| White, 301 | Colored, 4 | Total, 305 |

NATIVITY.

| | | | | | |
|---|---|---|---|---|---|
| Chicago | 126 | England | 9 | Prussia | 0 |
| Other parts U. S. | 52 | Germany | 42 | Scotland | 5 |
| Bavaria | 1 | Holland | 1 | Sweden | 5 |
| Bohemia | 5 | Ireland | 39 | Unknown | 3 |
| Canada | 8 | Isle of Man | 1 | | |
| Denmark | 2 | Norway | 6 | Total | 305 |

DEATHS BY SMALL-POX.
For the Month of April, 1868.

| | | | |
|---|---|---|---|
| 4th Ward | 1 | 12th Ward | 1 |
| 5th Ward | 3 | 15th Ward | 3 |
| 6th Ward | 1 | Lake Hospital | 2 |
| 7th Ward | 1 | | |
| 9th Ward Varioloid, | 1 | Total, | 35 |

MORTALITY BY WARDS FOR THE MONTH.

| Ward. | Mortality. | Pop. in 1866. | One death in | | Ward. | Mortality. | Pop. in 1866. | One death in | |
|---|---|---|---|---|---|---|---|---|---|
| 1 | 5 | 9,668 | 1,927 | 3-5 | 14 | 15 | 12,108 | 807 | 1-5 |
| 2 | 15 | 12,985 | 865 | 2-3 | 15 | 35 | 15,766 | 449 | 1-35 |
| 3 | 15 | 15,738 | 1,049 | 1-5 | 16 | 18 | 14,912 | 828 | 4-9 |
| 4 | 18 | 10,884 | 604 | 3-4 | County hosp. | 3 | | | |
| 5 | 19 | 9,610 | 495 | 1-4 | Chica. River, | 1 | | | |
| 6 | 13 | 10,680 | 813 | 11-13 | Bridewell, | 1 | | | |
| 7 | 43 | 18,755 | 436 | 1-6 | Home of the Friendl's, | 2 | | | |
| 8 | 14 | 10,429 | 745 | 0-0 | Marine hos. | 1 | | | |
| 9 | 19 | 13,940 | 733 | 4-7 | Mercy hos., | 3 | | | |
| 10 | 9 | 11,416 | 1,267 | 1-3 | St.Luke's hos. | 1 | | | |
| 11 | 16 | 12,924 | 807 | 3-4 | St.Jose. asyl. | 3 | | | |
| 12 | 21 | 12,695 | 604 | 1-2 | Lake Hosp., | 2 | | | |
| 13 | 13 | 8,188 | 629 | 11-13 | | | | | |
| Total, | | | | | | | | | 305 |

